# MT1-MMP as a Key Regulator of Metastasis

**DOI:** 10.3390/cells12172187

**Published:** 2023-08-31

**Authors:** Noritaka Tanaka, Takeharu Sakamoto

**Affiliations:** Department of Cancer Biology, Institute of Biomedical Science, Kansai Medical University, Hirakata 573-1010, Japan; tananori@hirakata.kmu.ac.jp

**Keywords:** MT1-MMP, cancer, metastasis, ECM

## Abstract

Membrane type1-matrix metalloproteinase (MT1-MMP) is a member of metalloproteinases that is tethered to the transmembrane. Its major function in cancer progression is to directly degrade the extracellular matrix components, which are mainly type I–III collagen or indirectly type IV collagen through the activation of MMP-2 with a cooperative function of the tissue inhibitor of metalloproteinase-2 (TIMP-2). MT1-MMP is expressed as an inactive form (zymogen) within the endoplasmic reticulum (ER) and receives truncation processing via furin for its activation. Upon the appropriate trafficking of MT1-MMP from the ER, the Golgi apparatus to the cell surface membrane, MT1-MMP exhibits proteolytic activities to the surrounding molecules such as extracellular matrix components and cell surface molecules. MT1-MMP also retains a non-proteolytic ability to activate hypoxia-inducible factor 1 alpha (HIF-1A) via factors inhibiting the HIF-1 (FIH-1)-Mint3-HIF-1 axis, resulting in the upregulation of glucose metabolism and oxygen-independent ATP production. Through various functions of MT1-MMP, cancer cells gain motility on migration/invasion, thus causing metastasis. Despite the long-time efforts spent on the development of MT1-MMP interventions, none have been accomplished yet due to the side effects caused by off-target effects. Recently, MT1-MMP-specific small molecule inhibitors or an antibody have been reported and these inhibitors could potentially be novel agents for cancer treatment.

## 1. Introduction

Matrix metalloproteinases (MMPs) are common proteinases composed of 23 members in humans, 6 of which were discovered as MMPs tethered to the plasma membrane, called membrane-type MMP (MT-MMP). Among MT-MMPs, MT1-MMP, also referred to as MMP-14, was the first MT-MMP to be identified [1] as a cell surface proteinase expressed in invasive tumors, followed by the discovery of MT2-MMP (MMP-15), MT3-MMP (MMP-16), MT4-MMP (MMP-17), MT5-MMP (MMP-24), and MT6-MMP (MMP-25). MT-MMPs are characterized as two subgroups, transmembrane (TM)-type and glycosylphosphatidylinositol (GPI)-type, which are tethered to TM and GPI, respectively. MT4-MMP and MT6-MMP are the GPI-type MMP and the rest of the MMPs are classified into TM-type. Although the functions of most of the MMPs are not well elucidated yet, MT1-MMP is the most well-characterized MMP in the aspect of its role in the invasion and metastasis of cancer cells, and even its non-proteolytic involvement in the indirect enhancement of hypoxia-inducible factor-1 alpha (HIF-1A) activity and the regulation of its endosomal trafficking. MT1-MMP is one of the collagenases and exhibits proteinase activities on extracellular matrix components, cell surface molecules, and certain types of cytosolic proteins. Now, MT1-MMP is known to be often associated with cancer metastasis in various mechanisms depending on the cell types. Regarding the extremely wide influence of MT1-MMP on various substrates and diseases, we would like to briefly summarize the involvement of MT1-MMP in cancer metastasis.

## 2. The Fundamentals of MT1-MMP

### 2.1. The Structure of MT1-MMP

**Figure 1:**
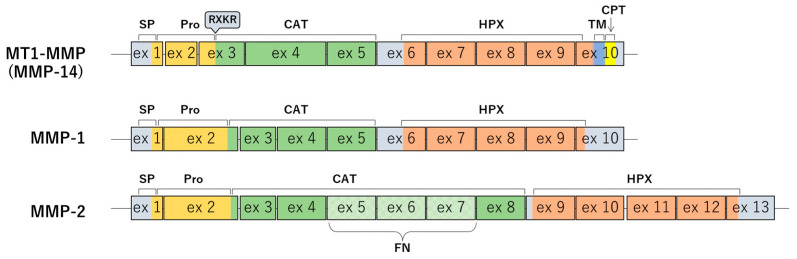
Exon mapping of MMP proteins. An exon mapping of MT1-MMP as a representative membrane type MMP is described compared with the MMP-1 as an original MMP and MMP-2 as an MMP that has a core relationship with MT1-MMP. SP: signal peptide, Pro: pro-domain, CAT: catalytic domain, HPX: HPX domain, TM: transmembrane domain, CPT: cytoplasmic tail, FN: fibronectin-like domain, RXKR: RXKR motif.

MMP families retain well-conserved domains within the extracellular region, which include signal peptide, pro-peptide, RXKR motif, and zinc-dependent catalytic motif within the N-terminal half; and the hemopexin-like (HPX) domain with adjacent linkers within the C-terminal half. What differentiates the TM-type and GPI-type is the TM domain with the adjacent cytoplasmic region, which comprises 20 amino acids, or the GPI signal domain, which lacks the cytoplasmic region, on the C-terminal end of the MT-MMPs, respectively [2,3]. Additionally, only TM-type MMPs retain the insertion of 8–9 amino acids named MT-loop within its catalytic domain [4,5]. MT1-MMP was the first MMP to be discovered as TM-type [1]. Although MT1-MMP is known as a cell surface protein, it initially localizes at the Golgi apparatus as an inactive state (pro-MMP) before it is transported to the cell surface and there, pro-MMP turns to the active state after receiving proteolytic processing between the RXKR motif and the catalytic domain via furin [6]. The *MT1-MMP* gene comprises 10 exons similar to most of the other MMPs except for MMP-2 and MMP-13 which comprise 13 and 8 exons, respectively. However, the domain coded with exon 2 in other MMPs is divided into two exons, exon 2 and 3, in MT1-MMP, and on the other hand, exon 3 and 4 in other MMPs converge on exon 4 only [7] (Figure 1).

### 2.2. How MT1-MMP Expressions Are Regulated

MT1-MMP is expressed in various types of cells such as fibroblasts, osteoblasts, and epithelial cells [4]. Additionally, cancer cells exhibit higher levels of MT1-MMP expression, and that associates with poor prognosis [8,9,10,11,12]. Since the distributions and the substrates of MT1-MMP are extremely diverse, its expression is strictly controlled at an appropriate level depending on the situation through transcriptional and epigenetic regulations. For example, the expression of MT1-MMP is maintained at a high level during mouse embryogenesis in the developing urinary bladder, skeletal formation, muscular tissues, and placenta [13]. The expression of MT1-MMP can also be upregulated along with that of MMP-2 upon the vascular damage and also the skin where wound repairing is occurring [14,15]. MT1-MMP not only with its structural feature but also with its gene regulation mechanism seems to be somewhat unique among MMPs although still little about the precise mechanism has been elucidated. Whereas the promoters of the other MMPs contain common TATA boxes and activator protein-1 (AP-1) binding sites, that of MT1-MMP lacks both a TATA box and AP-1 binding site, and possesses an Sp-1 binding site instead, located 92 nucleotides upstream of the transcription-initiating region [7]. The Sp-1 binding site is not completely the same as the classical sequences, but it still functions as a critical regulator of MT1-MMP expression as the deletion of this site abolishes its expression. In addition to Sp-1, other factors could regulate the transcription of MT1-MMP, which differs by the type of cells. These transcription factors include molecules such as Early Growth Response-1 (Egr-1), Nuclear Factor-kappa B (NF-kB), c-Myc, NK2 Homeobox (Nkx2), and p53. Of these transcription factors, the Egr-1 binding site partially overlaps with the Sp-1 binding site; thus, these two molecules compete with each other. In endothelial cells, Egr-1 rather than Sp-1 regulates the transcription of *MT1-MMP* [16]. As for the macrophages of the atherosclerotic plaque, Serum amyloid A-activating factor-1 upregulates MT1-MMP [17]. Whereas most of the transcription factors that bind to the MT1-MMP promoter function as the inducers of its expression, the interaction with p53 or PROX1 leads to the suppressive outcome [18,19,20]. The expression of PROX1 is shown to clinically correlate with that of MT1-MMP as well as a positive correlation with the survival rate in gastric cancer [19].

Epigenetic modifications are also common concepts that regulate gene expressions. As epigenetic alterations, the methylation of DNA, which can be frequently seen in CpG sites, generally suppresses the expression of tumor suppressor genes [21]. Core histones H2A, H2B, H3, and H4 can also receive several types of modifications, which are methylation, acetylation, phosphorylation, and ubiquitination [22]. The acetylation of histones is generally associated with the enhancement of gene expressions while methylation can function in both directions for up/downregulations [23]. These epigenetic modifications are frequently dysregulated in cancers. The promoter of *MT1-MMP* in breast cancer cells MCF-7, which lack MT1-MMP expression, are hypermethylated at CpG islands compared with other cancer cells with high MT1-MMP expression. Additionally, the trimethylation of H3K27 is hyper-upregulated. Therefore, the expression of MT1-MMP is also epigenetically regulated through DNA methylation and acetylation at H3K27 [24]. 

Recently, several microRNAs (miR) have been identified as the suppressor of MT1-MMP expression. Tang et al. revealed that epigenetic modification also contributes to the indirect inhibition of MT1-MMP expression mediated by EZH2, DNMT1, and miR-484, resulting in the suppression of the metastatic features of cervical cancers [25]. In this report, MT1-MMP is shown to be downregulated by miR-484, the expression of which is suppressed by the DNMT1-mediated methylation of its promoter. As the EZH2-stimulated upregulation of DNMT1 takes part in the methylation of the miR-484 promoter, the expression of MT1-MMP is also epigenetically regulated by the methylation of other factors in addition to the MT1-MMP promoter itself. In renal cancer cells (RCC), miR-485-5p, which retains the potential for inhibiting hepatocellular carcinoma and glioma development, is able to bind to MT1-MMP mRNA and suppress its expression [26,27,28]. The circular RNAs of the *patched-1* (*PTCH1*) gene (circPTCH1) counteract with miR-485-5p by impairing the interaction between miR-485-5p and MT1-MMP mRNA; therefore, circPTCH-1 elevates MT1-MMP expression and enhances the metastasis of RCC to the lung, liver, spleen, and colon. Similarly, miR-142-5p abrogates the expression of MT1-MMP in oral squamous cell carcinoma [29]. 

### 2.3. Intracellular Localization of MT1-MMP

When cancer cells invade the metastatic site, they generally utilize the cell surface protrusions, named invadopodia. Invadopodia are actin-based adhesive structures, in which the lipids are abundant, and associated with the proteolytic activity against the ECM components [30]. MT1-MMP is now regarded as a key regulator of invadopodia. The mechanism of the localization of the cell surface is known to be accomplished via several factors such as F-actin and vimentin [31,32]. Recently, vimentin, which is a common epithelial-to-mesenchymal transition (EMT) marker, is one of the factors that stimulate MT1-MMP in degrading the surrounding collagens of colorectal cancers. Vimentin takes part in functioning as one such stimulus for MT1-MMP activation [32]. Upon vimentin binding with myosin 10 (Myo10), Myo10 is aggregated at the tips of cell extensions. The formation of the Myo10–vimentin complex will then be associated with the addition of MT1-MMP in it, which facilitates the trafficking of MT1-MMP toward the cell surface and enhances the collagen proteolysis for ECM reorganization. Additionally, not just vimentin regulates the localization of MT1-MMP, as MT1-MMP could potentially regulate the expression of vimentin in contrast [33]. On the other hand, the MT1-MMP cytoplasmic tail-binding protein 1 (MTCBP-1) also binds to the cytoplasmic tail of MT1-MMP and interferes with the binding of MT1-MMP and F-actin [34,35,36]. The expression of MTCBP-1 abolishes the localization of MT1-MMP in the invadopodia at the cellular surface. After the cell surface localization of MT1-MMP, it is required to go through the series of dynamics of internalization and recycling to the invadopodia to sustainably keep its net amount of active form on the cell surface. For the internalization of MT1-MMP, it is processed in clathrin- and caveola-dependent manner [37,38,39]. Notably, not all internalized MT1-MMPs are recycled back to invadopodia as some of them experience autophagy-dependent degradation mediated by the interaction between the HPX domain of MT1-MMP and the cytoplasmic region of CD63 [40]. The mechanisms of recycling MT1-MMP into invadopodia have a lot of elusive points but the reported factors involved here are the components of soluble N-ethylmaleimide-sensitive factor attachment protein receptor (SNARE) proteins (vesicle-associated membrane protein, VAMP; Bet1), Rab GTPase, and sorting nexin (SNX) 27 [41,42,43,44,45]. VAMP3, VAMP7, and Bet1 are the three SNARE proteins that are reported to have contributed to MT1-MMP trafficking, which are all localized at the late (mature) endosomes, and depleting either of these molecules causes the failure of recycling to the invadopodia associated with the decreased proteolytic activity on ECM components. Retromers are heteromeric protein complexes that are composed of multiple vacuolar protein-sorting subunits and have an important role in the trafficking of transmembrane cargoes [46,47]. SNX family proteins support the recruitment of retromers on the endosomal proteins, thus regulating the endosomal recycling of transmembrane proteins. In certain breast cancer cells, SNX27 is associated with the proper trafficking of MT1-MMP to the cell surface and the defect in SNX27 function leads to the impairment of MT1-MMP-mediated ECM destruction [44]. 

### 2.4. MT1-MMP-Dependent Processing of Other MMPs

MMPs are the enzymes that function as zinc-dependent proteinases mainly against ECM components and some cell surface receptors. MT1-MMP was the first membrane-anchored type of MMP discovered as an activator of type IV collagenase, MMP-2 (also known as Gelatinase A), by processing pro-MMP-2 in invasive cancer cells [1]. The currently proposed mechanism for MMP-2 activation by MT1-MMP involves the ternary complex formed with MT1-MMP, pro-MMP-2, and tissue inhibitor of metalloproteinase-2 (TIMP-2). MT1-MMP homodimerizes through its HPX and TM domain. The N-terminal inhibitory domain of TIMP-2 binds to and inhibits the proteolytic activity of only one side of the dimerized MT1-MMP, which means the counterpart of dimerized MT1-MMP is free from TIMP-2. In addition to dimerized MT1-MMP, the C-terminal region of TIMP-2 also binds to the HPX domain of pro-MMP-2, thus ending up forming a ternary complex of dimerized MT1-MMP, TIMP-2, and pro-MMP-2 [48]. Based on the reports from Goldberg et al., pro-MMP is catalyzed by MT1-MMP and processed to the activated form (64 kDa form) upon proteolysis between residues N80 and Y81 with an intermediate product (68 kDa form) that was processed between residues N37 and L38 [49,50]. During this MT1-MMP-mediated proteolysis of MMP-2, TIMP-2 serves as a mediator in the ternary complex for the interaction of MT1-MMP and MMP-2. Of note, it seems the expression level of TIMP-2 should be well controlled as an insufficient nor excess amount of TIMP-2 suppresses the MT1-MMP-mediated proteolysis of MMP-2 [49]. Additionally, MT1-MMP also shows proteolytic activity against MMP-13, which is classified as type II collagenase [51]. The activation of pro-MMP-13 (60 kDa form) is achieved by the cleavage at its residues G84–Y85 producing 48 kDa form via the 56 kDa intermediate form initiated via the MT1-MMP activity [51,52]. Unlike MMP-2, the cleavage of MMP-13 proceeds independently of TIMP-2. 

### 2.5. ECM Components as Substrates of MT1-MMP

Although MMP-2 and MMP-13 can be activated through the cleavage of the pro-MMPs by MT1- MMP, the main substrates of MT1-MMP are the ECM components such as collagens, fibronectins, gelatins, and so on. ECMs are non-cellular and three-dimensional factors surrounding the resident cells of tissues, and their components are extremely versatile. More than a few hundred ECM-related molecules are regarded to exist; however, the core components are roughly classified into collagens, proteoglycans (e.g., heparan sulfate, keratan sulfate), and glycoproteins (e.g., fibronectin, laminins, elastin) [53,54]. In other ways, ECMs can be grouped into two forms, stromal matrix and basement membranes. The stromal matrix is diverse among the tissues and can even be reprogrammed within the same tissue in response to internal or external stress such as psychological stresses, physical injuries, and tissue regeneration [55]. The stromal matrix includes various types of collagens (except for collagen IV), fibronectin, and elastin; and the remodeling of these molecules involves the transformation of ECM stiffness and cell signaling, thus leading to various diseases including cancer progression [56]. On the other hand, the basement membrane is a structure with a relatively higher stiffness compared with the stromal matrix and it includes collagen IV, laminin, nidogen, agrin, and sulfate proteoglycans as key components [57]. The main role of the basal membrane is to form a stiff and well-organized surface of the tissues, which enables the separation from the surrounding environments and the homeostasis of tissues [58]. Tumor metastasis generally involves the remodeling of the basement membranes. MMPs are classified into subgroups depending on the preferential substrates: collagenase (MMP-1, -8, -13, -14, and -18), gelatinase (MMP-2 and -9), membrane types, and others (MMP-7, -12, -19, -20, -23, -26, and -28). Although some of the substrates identified for MMPs derive from in vitro experiments without showing the physiological interactions, MT-MMPs compared to the other soluble MMPs show a wider substrate spectrum. Since MT1-MMP has the widest specificity of substrates among MT-MMPs, MT1-MMP is regarded as the most important factor for ECM remodeling as MT-MMPs. The ECM-related substrates of MT1-MMP include collagen type I, II, and III, fibronectin, vitronectin, laminin-111, laminin-332, and so on [59,60,61,62,63]. Although MT1-MMP cannot process type IV collagen, which is a key component of basement membranes, MMP-2 can provide proteolytic processing to type IV collagen. Therefore, MT1-MMP can indirectly degrade type IV collagen via the activation of MMP-2. Consequently, MT1-MMP is capable of remodeling components of both the stromal matrix and basement membrane, thus possessing a key role in the invasion of cancer cells. 

### 2.6. Non-ECM-Related Substrates of MT1-MMP

Apart from ECM components and the MMP families, MT1-MMP exhibits proteolytic activities against cell surface molecules. Fibroblast growth factor receptors (FGFRs) are a family of receptor tyrosine kinases that are responsible for intra-membranous ossification during skull development and their activating mutations are sometimes related to tumor progressions [64]. A disintegrin and metalloprotease 9 (ADAM9) is a transmembrane protein that possesses a metalloproteinase domain but is distinct from the MMP family. ADAM9 can bind with FGFR2 and shed its juxtatransmembrane domain formed with 14 amino acid residues, thus leading to deactivation [65]. By MT1-MMP shedding ADAM9, it indirectly protects FGFR2 from cleavage by ADAM9 [66]. ADAM9 expression is negatively correlated with the malignancy level of prostate cancer. CD44 is a cell surface receptor that primarily binds with hyaluronic acids (HAs). MT1-MMP, through its HPX domain, binds with the cytoplasmic region of CD44 and cleaves it at the residues between R186 and G192 [60,67]. The importance of the interaction between MT1-MMP and CD44 is not just due to its influence on ECM structures but also to the determination of their localization to the cell surface [68]. As the cytoplasmic region of CD44 binds with cytoskeletal molecule F-actin, F-actin functions as a rail for trafficking MT1-MMP to the cell surface along with CD44 prior to the proteolytic modification of CD44 by MT1-MMP at the lamellipodia [68]. Recently, insulin receptor has been revealed to be digested with MT1-MMP, which causes obesity-induced insulin resistance along with aging [69].

### 2.7. Beyond Its Function as Proteinase

Hypoxia is one of the common features of cancer cells, especially invasive cells, as well as macrophages and a certain kind of stromal cells. HIF-1A is known as the key factor for adaptation to hypoxia by functioning as a transcription factor and upregulating the expression levels of adenosine triphosphate (ATP) production and genes related to angiogenesis such as vascular endothelial growth factor (VEGF). Under normoxic conditions, the activities of HIF-1 proteins are suppressed in two mechanisms, which are oxygen-dependent protein degradation and deactivation via factor-inhibiting HIF-1 (FIH-1). For the degradation of HIF-1, prolyl hydroxylases (PHDs) and an E3-ubiquitin ligase named von-Hippel Lindau (VHL) take responsible roles. As PHDs utilize oxygen molecules for their activity, the normoxic condition enables the hydroxylation of HIF-1A at residues P402 and P564 followed by proteasomal degradation upon recognition of HIF-1 hydroxylation via VHL. Therefore, the hypoxic condition increases the stability of HIF-1 and its activity through the cooperative function with p300/CBP. FIH-1 interferes with the interaction between HIF-1 and p300/CBP, which leads to the deactivation of HIF-1 without affecting the expression levels. In contrast, the Munc18-1-interacting protein (Mint3), which is also called amyloid beta precursor protein-binding family A member 3, enhances the activity of HIF-1. Mint3 is a protein that comprises a phosphotyrosine-binding domain and tandem PDZ domains (PDZa and PDZb) within the C-terminal half and with these domains, Mint3 serves as an adaptor protein. The binding partners of Mint3 also include furin [70]. On the other hand, the N-terminal region of Mint3, which is intrinsically disordered, especially residues G77–G88, was discovered to form dimers with FIH-1. This interaction of Mint3 and FIH-1 indirectly activates HIF-1 by suppressing the binding of FIH-1 and HIF-1. MT1-MMP with its cytoplasmic region was identified to bind with FIH-1 and also mediate the colocalization of FIH-1 and Mint3 [71]. The cytoplasmic region of MT1-MMP, especially amino acid residues Q575 through L578 (QRSL), has the key responsibility for binding with FIH-1 since the deletion of CPT or the mutants of QRSL diminished the MT1-MMP/FIH-1 interaction. Not only that, FIH-1, which generally colocalizes with Mint3 at the perinuclear region, shows diffused expression within the cytoplasm, losing the ability to form a complex with Mint3 when the CPT of MT1-MMP is defective. Conclusively, we have identified MT1-MMP as another factor that indirectly upregulates HIF-1 activity [72].

## 3. The Impact of MT1-MMP on Cancer Metastasis

### 3.1. ECM Remodeling by MT1-MMP

Metastasis involves complex and multiple steps of the cellular journey such as intravasation, extravasation, and localization at the metastatic sites. As MT1-MMP functions as a collagenase which directly sheds type I–III collagen, or indirectly type IV collagen through the activation of MMP-2, MT1-MMP is indirectly involved in the destruction of basement membrane structures, which enables cell invasion. Weise et al., using an established breast cancer cell line, proved the wide contribution of MT1-MMP as well as MT2-MMP to the invasive activity of cancer cells into vessels and basement membranes with their proteolytic activity against collagens, leading to the degradation of ECMs [73]. As the direct contribution of MT1-MMP on extravasation is not well elucidated, the stable formation of invadopodia by its structural components Tks4/5 and cortactin is a prerequisite feature for extravasation around the extravascular region of murine lung and several cancer cells such as HT-1080 (fibrosarcoma), B16F10 (murine melanoma), MDA-MB-231LN (breast), and T24 (bladder), which share similar phenotypes [74]. As invadopodia indeed include MT1-MMP, as already stated above, MT1-MMP may be the actual executor of invadopodia-dependent extravasation.

However, the stimulus that activates the MT1-MMP activity varies. Although the functions of CD44 are so complicated, since they cover a wide range of physiological processes, they are generally associated with the progression and metastasis of tumors. One of their common reasons for increasing malignancy is that the binding of HA with CD44 can activate the key oncologic signaling cascade such as the mitogen-activated protein kinase (MAPK) and Phosphatidyl inositol triphosphate kinase (PI3K)/Akt pathways [75]. In addition, several reports implicated that CD44 shedding is associated with the migration or metastasis of tumors. Various cancer cell lines (e.g., lung, sarcoma, pancreas) exhibit enhanced migration upon the cleavage of CD44 by MT1-MMP [60,68]. Consistently, low-molecule HA fragments (HA oligosaccharides) also induce the stimulus for CD44 cleavage, resulting in the elevated migration of pancreatic cancer cells [76]. As the internal cytoplasmic domain of CD44 (CD44-ICD) released from the cell surface in turn functions as a transcription factor, it could potentially upregulate the expressions of genes required for migration including CD44 itself [77]. ALIX has recently been discovered to upregulate MT1-MMP and MMP-9 expression and the depletion of ALIX attenuates the invasive/migrative activity of head and neck cell carcinoma (HNSCC) cells [78].

Cleavage of ECM molecules in some cases releases bioactive fragments like growth factors, cytokine/chemokines, and the shorter fragments of ECM components which often comprise cytokine/chemokine-like structures, thus termed matrikines. Transforming growth factor beta (TGF-β) is a well-known factor that is involved in angiogenesis and metastasis. Latent TGF-β is predominantly bound to latent TGF-β binding protein-1 (LTBP-1) [79]. Upon the proteolysis of LTBP-1 in endothelial cells, latent TGF-β is released into the ECM environment and supports tumor progression and metastasis. MMP-2 is also able to produce elastin-derived matrikines composed of specific peptides (VGVAPG or AGVPGLGVG) and they were revealed to promote tumor progression [80,81]. In this case, MT1-MMP is indirectly involved in releasing elastin-derived matrikines through the direct activation of MMP-2. Laminin-332, which is composed of α3, β3, and γ2 chains, is also one of the ECM components that release matrikines upon cleavage via MT1-MMP and MMP-2 [62,63,82]. MT1-MMP cleaves and produces domain III fragment (DIII) within γ2 chain and DIII fragments, which is revealed to induce the EGFR-dependent migrative activity of breast cancer cells with its EGF-like structure [83]. Not only γ2 but also the α3 chain with its LG3 module contributes to the enhanced cell adhesion and migration associated with α3β1 integrin [84]. Conclusively, MT1-MMP has a wide contribution to remodeling the metastatic niche degrading the ECM and adhesion molecules, and in some cases, proteolytically producing the bioactive molecules in the secondary localization site for cancer metastasis.

### 3.2. The Activation of Oncogenic Signaling by MT1-MMP

As one of the mechanisms of MT1-MMP-derived tumor invasion, Be¨liveau et al. reported the activation of the MAPK cascade. According to the report, excess cellular expression of MT1-MMP induces the upregulation of phospho-ERK (p-ERK); however, the effect requires both the intact cytoplasmic region and the activity of MT1-MMP [85]. Notably, the activation of ERK mediated via MT1-MMP is dependent on serum and the direct contact of MT1-MMP with ECM molecules including at least either fibronectin, type I collagen, or gelatin. Conversely, Mignatti et al. had reported a similar case of the MT1-MMP-dependent activation of the MAPK cascade; however, they proposed the non-proteolytic mechanism of p-ERK activation that enhances the cell proliferation and migration as well as tumor growth [86]. This non-proteolytic mechanism also requires the existence of the cytoplasmic region of MT1-MMP, especially residues Y573–R575, showing the critical role and the direct contact of MT1-MMP and TIMP-2 through either or both the catalytic and HPX domain of MT1-MMP. Y573 in MT1-MMP is phosphorylated through the activity of Src tyrosine kinase and the modification causes the sphingosine-1-phosphate-induced migration [87]. Interestingly, the protein expression of MT1-MMP with an intact residue Y573 induces the phosphorylation of Src (Y416), which is an activating modification, independently of the proteolytic activity of MT1-MMP [88]. In total, MT1-MMP and Src are mutually supportive in directing cell proliferation and migration. Several other groups also reported that MT1-MMP mediated cell growth and migration depending on its proteolytic activity, so there remains further discussion for the requirement of the proteolytic activity of MT1-MMP for cell growth and migration. Since TIMP-2 can function bidirectionally against the activation of MMP-2 depending on its concentrations, a similar case could be applied for this case, and a properly controlled concentration of TIMP-2 could be the clue for clarifying the discrepancy since Mignatti et al. is the only group that used a relatively low concentration of TIMP-2, which is close to the physiological concentration, and demonstrated the independency of the proteolytic activity of MT1-MMP [89,90,91]. PI3K/Akt signaling is also a common pathway that is related to cancer malignancy in various aspects. TIMP-2 is also capable of activating PI3K/Akt signaling in the presence of MT1-MMP but does not require its proteolytic activity [92]. For the TIMP-2- and MT1-MMP-dependent activation of the PI3K/Akt cascade, the involvement of the cytoplasmic region of MT1-MMP is yet to be elucidated. Although FGFR and the downstream RAS activity are one of the molecules that retain the potential for activating both the MAPK and PI3K/Akt cascades, the TIMP-2-mediated induction of MAPK signaling was the only one that was dependent on the FGFR activity [92].

### 3.3. Metastasis Promotion via the Non-Proteolytic Activity of MT1-MMP

MT1-MMP occasionally demonstrates non-proteolytic involvement in cell invasion with the existence of its cytoplasmic region. The residues from L571 through C574, which reside in the cytoplasmic region, are critical sites that regulate the internalization of MT1-MMP [93]. The depletion of the cytoplasmic region disables MT1-MMP to internalize without affecting its proteolytic activity; however, it also abrogates the migrative ability of cancer cells, proving that cancer cells employ the cytoplasmic region but not the proteolytic ability of MT1-MMP in certain circumstances. One possible factor that is involved in this proteolytic activity-independent migration is the palmitoylation at C574 [94]. Interestingly, MT1-MMP in endothelial cells shows its localization where intercellular adhesion occurs in an ECM-type-specific manner. This cell surface localization is stimulated by type I collagen, fibronectin, or fibrinogen surrounding the endothelial cells and is mediated by the interaction between the cytoplasmic region of MT1-MMP and either β1 or αvβ3 integrin [95]. Importantly, these ECMs abrogated both the internalization and the proteolytic activity of MT1-MMP but increased the migrative activities. This unique relationship between endothelial MT1-MMP and ECMs may be involved in regulating the angiogenesis or tumor invasion into the tissue. Recent reports from Coppolino et al. made it firm that invadopodia formation and the internalization of MT1-MMP require the activity of β1 activity and the subsequent activation of Src and EGFR for the phosphorylation of the MT1-MMP cytoplasmic region [96,97]. 

**Figure 2:**
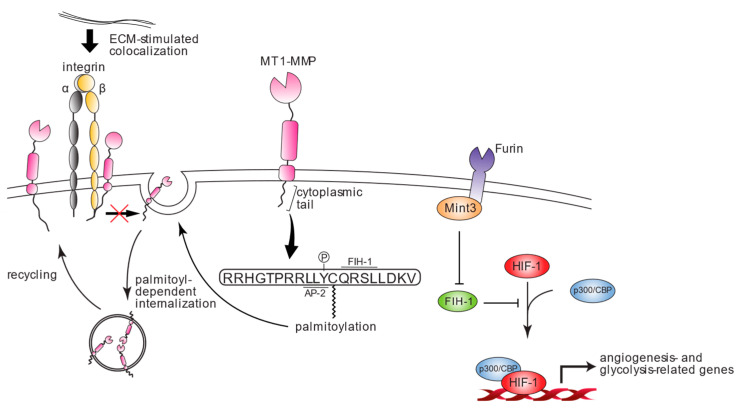
Schematic non-proteolytic function of MT1-MMP. Non-proteolytic function of MT1-MMP is mainly regulated via its cytoplasmic region. The internalization and recycling circuit of MT1-MMP required for its intact proteinase activity is mediated via the palmitoylation at C574 residue. On the other hand, ECMs such as type I collagen induce the cell surface colocalization of MT1-MMP and integrin subunits, which is also bound with the cytoplasmic region of MT1-MMP. Under intercellular contacts, this complex impairs the internalization of MT1-MMP. Additionally, FIH-1 bound at the cytoplasmic region of MT1-MMP lacks its ability to bind with HIF-1; thus, it means MT1-MMP is indirectly involved in upregulating HIF-1 activity.

Other than the involvement of MT1-MMP in extracellular environments, it can also affect intracellular molecules. As stated above, the cytoplasmic region of MT1-MMP binds to FIH-1, which leads to the activation of Mint3 associated with HIF-1A activation. This MT1-MMP-mediated HIF-1A activation via the FIH-1–Mint3–HIF-1A axis was initially discovered as an ATP production mechanism in macrophages [72,98]. However, cancer cells were also identified to utilize a similar mechanism for their ATP production through rapid glucose metabolism, named the Warburg effect [99]. Since Mint3 has the potential for promoting cell proliferation and metastasis in various types of cancer cells (e.g., breast cancer, MDA-MB-231; fibrosarcoma, HT-1080; epidermoid carcinoma, A431; non-small cell lung cancer, A549; and urothelial carcinoma, RT-112), the activity of MT1-MMP is also responsible for exhibiting these features of cancer cells [100,101,102] (Figure 2). Additionally, the MT1-MMP-mediated induction of the HIF-1A activity in the hematopoietic stem cell is involved in releasing secretory factors like kit ligand and erythropoietin, and chemokines such as stromal cell-derived factor-1 and interleukin-7 in addition to VEGF as a canonical transcription target of HIF-1A [103]. Although this finding is related to hematopoietic homeostasis, it could also affect the metastasis which involves the vascular niches.

## 4. Negative Regulators of MT1-MMP

### 4.1. Endogenous Negative Regulators of MT1-MMP

Since the MT1-MMP affects the migration/invasion ability of cancer cells either with its proteolytic or non-proteolytic activity, the inhibition of its activity could potentially abrogate the cancer malignancy. The activity of MT1-MMP can be physiologically regulated through the inhibition of its expression, direct inhibition of its catalytic domains, or its trafficking among the Golgi apparatus, ER, and transmembrane localization. The appropriate amount of TIMP-2 functions as a bridge between MT1-MMP and MMP-2, and supports the activation of MMP-2. However, the excess amount of TIMP-2 rather suppresses the proteolytic activity of MT1-MMP since it could bind to both sides of the MT1-MMP dimers, resulting in the inhibition of the gelatinolytic activity of MMP-2 [104]. TIMP-2 additionally inhibits FGF-2-induced endothelial cell proliferation, mitogenic activity of epidermal growth factor, and angiogenesis by increasing a protein tyrosine phosphatase activity associated with FGF and VEGF receptors [105,106,107]. Therefore, TIMP-2 suppresses metastasis through proteolytic- and growth factor-dependent mechanisms. Reversion-inducing-cysteine-rich protein with Kazal motifs (RECK) also functions as a tumor invasion suppressive protein by inhibiting the proteolytic activity of MT1-MMP and MMP-2 in addition to its influence on MMP-9 expression [108,109]. As one of the factors that negatively regulate the expression of MT1-MMP, p53 tumor suppressive protein, which is reverted by interleukin-6, was reported by Cao et al. [20]. Schlafen 5 (SLFN5) is a protein which was discovered to have an inhibitory effect on cell motility and invasiveness in an interferon alpha-dependent manner in renal cancer and melanoma cells [110,111]. One of the underlying mechanisms of the SLFN5-dependent inhibition of cancer invasions, SLFN5 suppresses the expression of MT1-MMP through the inhibition of Akt activity and the subsequent activation of glycogen synthase kinase 3 beta and the deactivation of β-catenin, which is an executor transcription factor in the Wnt/β-catenin cascade [112]. 

WD Repeat and FYVE Domain Containing 2 (WDFY2) is a protein which was discovered to localize at the tubular structure of endosomes for the first time by Sneeggen et al. [113]. Whereas vesicles with VAMP3 SNARE protein can transport MT1-MMP to the plasma membrane, WDFY2 tethered to the phosphatidylinositol 3-phosphate which localized at the same VAMP3 positive vesicles interferes with the VAMP3-dependent recycling of MT1-MMP into invadopodia [45,113,114]. WDFY2 functions by disrupting the endocytosis-recycling circuit of MT1-MMP and abrogates the invasion of cancer cells into collagen-rich ECMs. 

Breast cancer can be classified into non-/pre-invasive types called ductal carcinoma in situ (DCIS) and invasive breast cancer (IBC). To date, non-metastatic cells 1 (NME1) is the latest protein molecule to be identified as a suppressor of MT1-MTP-driven metastasis. The expression levels of NME1 and MT1-MMP are revealed to negatively correlate and additionally, NME1 levels switch from high to low while MT1-MMP switch from low to high upon transforming from DCIS to IBC [115]. NME1 drives the endocytosis of MT1-MMP and destructs the balance of internalization of recycling during breast cancer stays as DCIS, which could potentially result in the lysosomal destruction of MT1-MMP. Overexpression of NME1 leads to the decreased invasive activity of breast cancer cells. Of note, the similar correlation between NME1 and MT1-MMP also applies to other cancers including colorectal, endometrial, ovarian, prostate, and HNSCC tumors [115]. 

### 4.2. Artificial Inhibitors of MT1-MMP

The cumulative evidence of MT1-MMP enhancing tumor progression prompted people to spend efforts developing chemical inhibitors of MT1-MMP. The initial clinical attempts to overcome MT1-MMP-driven tumor malignancy started in the 1990s [116]. The original concept of MT1-MMP inhibition was through depriving zinc ions of MT1-MMP by zinc chelators (e.g., Batimastat, BB-94; Marimastat; BB-2516). However, their off-target inhibition of the ADAM family protein led to severe adverse events, especially musculoskeletal syndrome (MSS), and the clinical trials of these inhibitors had to be terminated [117,118]. Even with the inhibitor with a similar mechanism of action and higher selectivity to MMPs (e.g., tanomastat, prinomastat, rebimastat), their clinical trials still were not successful due to the other severe side effects such as bone marrow suppression and venous thromboembolism despite improved MSS [119]. Recently, quinoline derivatives, clioquinol, and chloroxine were identified as novel MT1-MMP inhibitors. These compounds were discovered through the screening of the proteolytic activity on VEGFR peptides. Both of these compounds bind to the tunnel structure that resides in the Ω-loop region of the catalytic domain [120]. While chloroxine shows affinity against MMP-9 and MMP-13 as well, clioquinol is highly specific to MT1-MMP. Although the actual inhibitory effect of these quinoline derivatives on cancer cell invasion or metastasis is not validated, at least they impair the proteolytic activity of MMP-2 mediated by MT1-MMP. DX-2400 is an antibody that selectively binds to MT1-MMP and inhibits its activity associated with the subsequential inhibition of MMP-2 activation [121]. Although DX-2400 has yet to be forwarded in clinical trials, it shows significant efficacy in the suppression of breast cancer cell metastasis, both in vitro and in vivo [121]. So far, the latest antibody agent for MT1-MMP identified is IgG3369. This antibody also effectively exhibits the inhibitory effect of tumor metastasis in the mouse xenograft model of triple-negative breast cancers [122].

## 5. Conclusions

MT1-MMP, which was initially identified in the invasive front of cancer cells, has long been studied with the aim to elucidate its contributions to tumor metastasis. Although a complete understanding of the features of MT1-MMP is complex due to its diverse effects on ECMs, cell surface molecules, or intracellular signaling via its proteolytic and non-proteolytic activities, it has become a firm fact that MT1-MMP promotes tumor malignancy through its influence on tumor progression, metastasis, and angiogenesis. Until now, doxycycline is the only Food and Drug Administration-approved MMP inhibitor, which has not been proven to inhibit MT1-MMP. The past failures in the clinical trials of MT1-MMP inhibitors are mainly due to off-target-driven side effects but highly MT1-MMP-specific inhibitors have recently been reported from multiple groups so they could be a potential intervention that could conquer the MT1-MMP-driven tumor malignancy. 

On the other hand, we have to keep in mind that MT1-MMP is also required in certain situations such as embryogenesis and the maintenance of macrophages. Therefore, MT1-MMP inhibitors should be administrated with precise care. Under these cautions, MT1-MMP inhibitors could potentially be promising agents that induce various advantages not just for cancer treatments but also for other diseases including rheumatoid arthritis, age-associated diabetes, and atherosclerosis.

## Data Availability

Not applicable.
